# Case report: Milk oral immunotherapy for adult-onset cow’s milk-induced anaphylaxis

**DOI:** 10.5415/apallergy.0000000000000218

**Published:** 2025-12-02

**Authors:** Jae-Hyuk Jang, Sooyoung Lee, Young-Min Ye

**Affiliations:** 1 Department of Allergy and Clinical Immunology, Ajou University School of Medicine, Suwon, Korea; 2 Department of Pediatrics, Ajou University School of Medicine, Suwon, Korea

**Keywords:** Adult, cow’s milk, food allergy, IgE, immunotherapy

## Abstract

While food allergies affect individuals across all age groups, oral immunotherapy (OIT) has been widely studied and implemented in pediatric populations. Here, we report a successful case of cow’s milk (CM) OIT in a 60-year-old male with adult-onset cow milk allergy. The patient who was previously tolerated with CM initially presented with generalized pruritus, rash, dyspnea, urticaria, and dizziness occurring 4 hours after consuming milk-containing foods. The patient was positive on the skin prick test and for specific IgEs to milk (24.4 kU/L) and casein (31.2 kU/L). After diagnosis, CM OIT was initiated using bread containing milk protein. The protocol began with a 1/4 slice of milk bread (1.55 g of milk protein) and gradually increased to 1 full slice (6.2 g of milk protein) over the course of 4 years. Throughout the period, the patient’s immunological markers consistently improved, with his CM-specific IgE level decreasing from 24.40 to 1.50 kU/L and his casein-specific IgE level decreasing from 31.20 to 2.24 kU/L. The patient successfully completed an oral food challenge with 190 mL of cow milk and currently maintains a daily consumption of 200 mL of CM without adverse reactions. This case demonstrates that CM OIT can be successfully implemented in elderly patients with adult-onset CM allergy. The clinical and immunological responses suggest that immune modulation remains possible even in older adults, suggesting potential therapeutic options for adult-onset food allergies.

Food allergies represent a significant public health concern, with the increasing prevalence affecting individuals across all age groups, not just children and adolescents [1]. Adult food allergies often persist and can significantly impact quality of life, with reactions potentially being more severe than those in children [2]. The prevalence of cow’s milk allergy varies widely across studies, ranging from 0.3% to 2.5% in pediatric populations and less than 0.5% in adults [2, 3].

Oral immunotherapy (OIT) has emerged as a promising treatment for food allergies, with extensive research and clinical experience in pediatric populations, particularly for peanut and cow’s milk (CM) allergies [4]. However, despite the demonstrated success of OIT in children, its application in adult patients remains limited [5]. While several studies have shown the efficacy of peanut OIT in children, with success rates ranging from 60% to 80%, research on OIT in adults is sparse, particularly for CM allergies [5, 6]. The limited availability of data on OIT in adults poses a significant challenge in clinical practice. This case report aims to present the evidence regarding the feasibility and effectiveness of CM OIT in adult patients.

A 60-year-old male with no underlying diseases presented with systemic symptoms, including generalized pruritus, rash, urticaria, dyspnea, and dizziness, that developed 4 hours after consuming dairy products. Following the initial episode in July 2020, the patient exhibited reproducible symptoms that consistently developed within 60 minutes after eating various dairy-containing foods, including coffee containing milk, yogurt, and milk-marinated pork, on separate occasions. All symptomatic cases required emergency department care. He tolerated wheat-based foods without any symptoms.

On the allergy skin test, the patient showed a positive response only to CM and a negative response to common aeroallergens and other food allergens. Initial laboratory tests revealed an elevated serum total IgE level (354 kU/L) and positivity for the specific IgEs to CM (24.40 kU/L) and casein (31.20 kU/L). The tests were negative for specific IgEs to wheat (0.32 kU/L) and gliadin (<0.05 kU/L), as were those for other allergens, including house dust mites and α- and β-lactoglobulin. Specific levels of IgG4 to casein were not assessed at the initial visit. The serum level of 25-(OH) vitamin D was 13.3 ng/mL. Since the elimination of CM from his diet, the patient has not experienced any symptoms. The patient was diagnosed with adult-onset CM allergy, and due to the recurrent nature of the symptoms, we decided to proceed with OIT. To determine the tolerable dose of CM for OIT, an oral food challenge was conducted using commercially available CM bread (total weight 400 g, containing 62 g of CM protein, with 6.2 g of CM protein per slice) (Fig. 1). Starting with 1/16 of a slice at 15-minute intervals, the dose was escalated to 1/2 slice, at which point the patient developed immediate hypersensitivity symptoms, including pruritus and throat tightness. OIT (Supplementary Table S1, https://links.lww.com/PA9/A66) was initiated with a quarter slice of CM bread daily and continued for 3 months, accompanied by a vitamin D supplementation (2000 IU daily).

**Figure 1.**
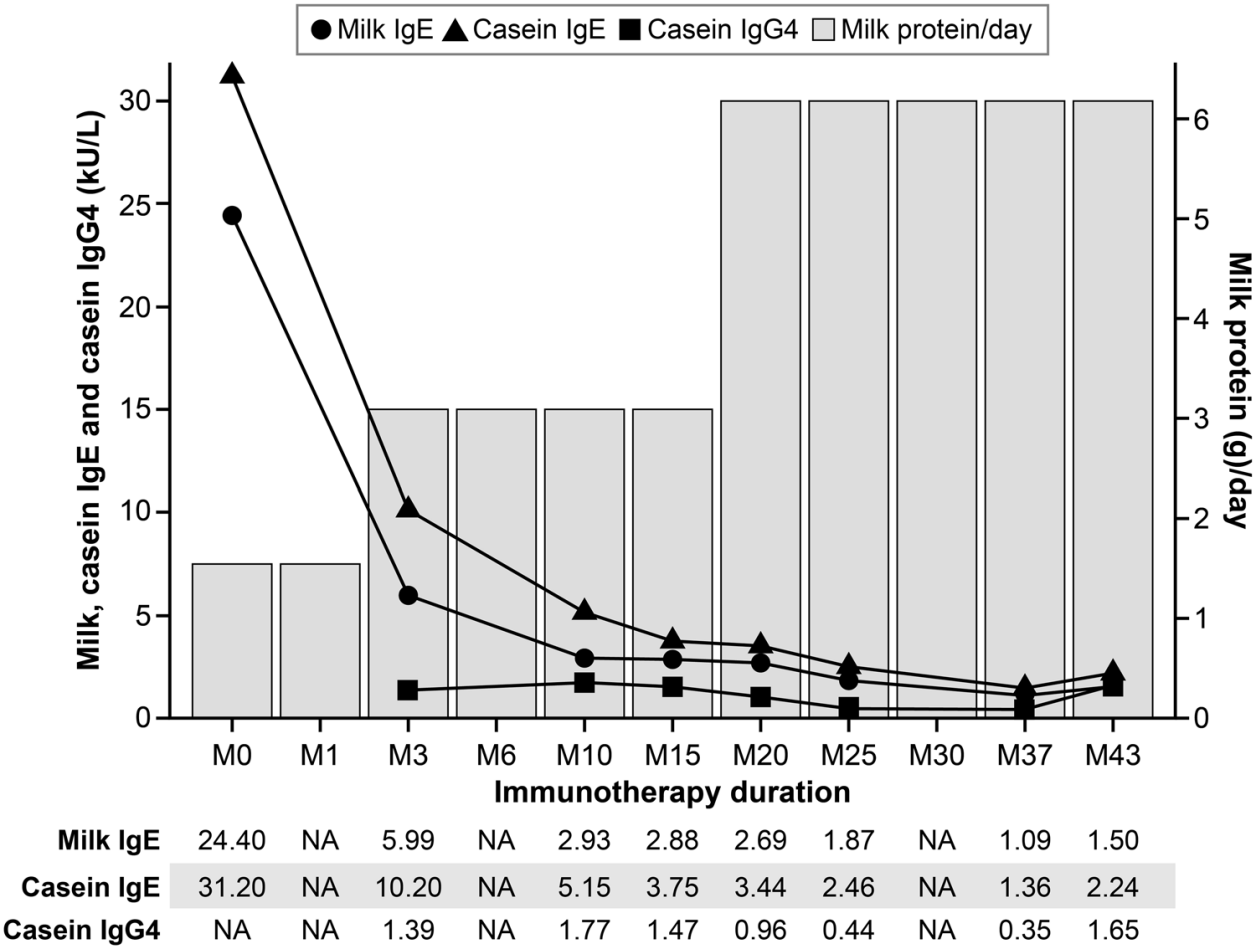
Changes in daily milk protein doses and serologic markers during cow’s milk oral immunotherapy

Three months after starting OIT, the dose was increased to a half slice. One year after maintaining OIT, the patient tolerated CM doses of up to 1.5 g. After 3 years of OIT, the patient successfully passed an oral food challenge with 5.7 g of CM without adverse reactions, after which the daily consumption of 200 mL of CM was recommended.

The patient’s total serum IgE level slightly decreased to 194 kU/L by January 2022. His CM-specific IgE levels substantially decreased from an initial value of 24.40 kU/L at baseline to 5.99 kU/L by June 2021. Similarly, casein-specific IgE levels markedly decreased from 31.20 kU/L at baseline to 10.20 kU/L in Jun 2021. His CM- and casein-specific IgE levels steadily decreased but slightly increased after Oct 2024. The casein-specific IgG4 level fluctuated throughout the treatment period without any clear association with the patient’s tolerance of CM. The level initially increased from 1.39 mg/L to 1.77 mg/L in Jan 2022, then decreased to 0.35 mg/L in April 2024, before increasing again to 1.65 mg/L in Oct 2024 (Supplementary Table S2, https://links.lww.com/PA9/A66). As of Oct 2024, the patient was able to consume CM-containing beverages, including lattes, without any symptoms. He maintains a daily CM intake with continued outpatient monitoring. The patient agreed to the publication and signed the informed consent document.

The current guidelines for managing CM allergies primarily recommend the strict avoidance of CM products or, in cases of IgE-mediated reactions, the preventive use of anti-IgE therapies [7]. However, these approaches fail to address the significant psychosocial impact of food allergies. Dietary restrictions can substantially affect an individual’s quality of life, not only in adolescents but also in adults, causing stress and anxiety in social and daily activities. OIT offers a potential solution by enabling patients to safely consume problematic foods, thereby reducing the psychological burden of food avoidance [8].

The successful implementation of CM OIT in this elderly patient expands upon previous research primarily focused on pediatric populations [8]. While studies have demonstrated OIT efficacy in adults over 18 years [5, 9], this case demonstrates successful CM OIT in a patient aged ≥60 years. Despite CM OIT’s higher risks [6], our patient achieved desensitization through a 4-year protocol without serious events, suggesting age alone should not exclude patients.

A recent meta-analysis demonstrated that children with vitamin D insufficiency are more likely to have food allergy episodes and multiple food sensitivities [10]. Notably, no studies have evaluated the relationship between vitamin D deficiency and food allergy in adults. In our patient, vitamin D deficiency was diagnosed at the initial visit. Given the immunoregulatory effects of vitamin D, daily supplementation with 2000 IU of vitamin D was maintained throughout the OIT. Therefore, measuring serum vitamin D levels is important for both pediatric patients with food allergies and adult patients. However, the beneficial role of vitamin D supplementation during OIT in adults with food allergies remains to be verified.

In conclusion, this case highlights that OIT can be a viable treatment option for adult-onset CM allergy, even in elderly patients. The potential benefits of OIT extend beyond simple allergen tolerance, offering improvements in quality of life in adult patients.

## Acknowledgement

This work was supported by a grant from the National Research Foundation of Korea funded by the Korean government (NRF-2022R1A2C2006607).

## Supplementary material

Supplementary Tables S1 and S2 can be found via https://links.lww.com/PA9/A66

Supplementary Tables S1 and S2

Click here to view

## Supplementary Material
