# Supplementary material for: Case report: Milk oral immunotherapy for adult-onset cow’s milk-induced anaphylaxis
Source: Asia Pac Allergy. 2025 Dec 2;15(4):343–5. doi: 10.5415/apallergy.0000000000000218 (PMC12672181; doi:10.5415/apallergy.0000000000000218)
Supplement: Supplementary file 1 [file pa9-15-343-s001.pdf]

**Supplementary material**

**Table S1**. Oral immunotherapy protocol

| Months of oral immunotherapy | Slice of bread | Weight (g) | Cow's milk protein amount (g) |
|---|---|---|---|
| 1day | 1/4 | 10 | 1.55 |
| 1 | 1/4 | 10 | 1.55 |
| 3 | 1/2 | 20 | 3.1 |
| 6 | 1/2 | 20 | 3.1 |
| 10 | 1/2 | 20 | 3.1 |
| 15 | 1/2 | 20 | 3.1 |
| 20 | 1 | 40 | 6.2 |
| 26 | 1 | 40 | 6.2 |
| 32 | 1 | 40 | 6.2 |
| 38 | 1 | 40 | 6.2 |
| 44 | 1 | 40 | 6.2 |

The bread used in this case contains 15.5% milk per loaf, which is currently not on sale.

**Table S2**. Changes in specific IgE and IgG4 levels throughout immunotherapy

| | Reference | Baseline | Mar 2021 | Jun 2021 | Dec 2021 | May 2022 | Oct 2022 | Apr 2023 | Apr 2024 | Oct 2024 |
|---|---|---|---|---|---|---|---|---|---|---|
| Total IgE (kU/L) | 0-114 | 354 | NA | 304 | 194 | NA | NA | NA | NA | NA |
| Milk IgE (kU/L) | 0-0.35 | 24.40 | NA | 5.99 | 2.93 | 2.88 | 2.69 | 1.87 | 1.09 | 1.50 |
| Casein IgE (kU/L) | 0-0.35 | 31.20 | NA | 10.20 | 5.15 | 3.75 | 3.44 | 2.46 | 1.36 | 2.24 |
| Casein IgG4 (mg/L) | | NA | NA | 1.39 | 1.77 | 1.47 | 0.96 | 0.44 | 0.35 | 1.65 |
| Alpha Lactalbumin IgE (kU/L) | 0-0.35 | <0.05 | | | | | | | | |
| Beta Lactoglobulin IgE (kU/L) | 0-0.35 | <0.05 | | | | | | | | |

NA, non-applicable
